# Differences in parasitism of root-knot nematodes (*Meloidogyne* spp.) on oilseed radish and oat

**DOI:** 10.21307/jofnem-2020-043

**Published:** 2020-04-24

**Authors:** Negin Hamidi, Abolfazl Hajihassani

**Affiliations:** 1 Department of Plant Pathology, University of Georgia, Tifton, GA 31793

**Keywords:** Host response, histopathology, oilseed radish, oat, root-knot nematodes, resistance

## Abstract

Oilseed radish and oat are cool season annual crops that are potentially used as “trap” or “biofumigant” crops for the suppression of plant-parasitic nematodes in soil. Cultivars of oilseed radish (Carwoodi, Cardinal, Final, Image, Concorde, Control, Eco-Till, Karakter and Cannavaro), white (Tachiibuki) and black (Pratex) oats were evaluated for their ability to reduce reproduction of three root-knot nematode species: *Meloidogyne javanica*, *M. incognita* race 3, and *M. arenaria* race 1. Nematode penetration and development were also evaluated using selected resistant and susceptible cultivars under greenhouse conditions. Root galling severity, number of eggs per gram of fresh root, and rate of reproduction varied among the cultivars in response to nematode infection. Oilseed radish cv. Carwoodi was resistant to *M. javanica*, whereas Karakter and Concorde were maintenance hosts allowing the nematode to maintain or increase its population on the plants. For *M. incognita*, Control and Carwoodi oilseed radish and Tachiibuki oat were resistant hosts. The cultivars that supported little reproduction of *M. arenaria* were Karakter and Carwoodi radish, and Tachiibuki oat. Comparable numbers of nematodes entered the roots of susceptible and resistant cultivars of oilseed radish and oat during early stages of infection. However, the development of the nematodes as evident from counting young and egg-laying females in roots were significantly decreased or inhibited in the resistant cultivars compared to the susceptible cultivars indicating that resistance occurs at post-infection stages. Histopathological examinations of galled-root tissues also revealed the susceptibility and resistance responses of selected cultivars of oilseed radish and oat to these nematode species.

Root-knot nematodes (*Meloidogyne* spp.) are obligate, sedentary endoparasites that can infect both the below- and above-ground parts of many crops at different developmental stages. *Meloidogyne* spp. are serious pathogens of multiple vegetable crops in the southern United States and worldwide (Johnson et al., 1992; Jones et al., 2013; Hajihassani, Davis, and Timper, 2019; Hajihassani, Rutter, and Luo, 2019). In Georgia, more than 66% of vegetable growing areas are infested with at least one species of root-knot nematodes at the level of one nematode per 100 cm^3^ of soil where management is needed to achieve economically reasonable yields (Marquez and Hajihassani, 2019). The nematode species most associated with vegetable production in Georgia are *M. incognita*, *M. arenaria*, and *M. javanica* (Marquez et al., 2019).

The life cycle of root-knot nematodes is completed in three basic stages: egg, juvenile (J), and adult stages. Eggs are deposited in a gelatinous mass that protects eggs from environmental extreme (e.g. high temperature) and microbes. The egg masses can be found on the surface of galled roots or embedded within the gall tissue. Under favorable conditions, egg development takes place and the first stage juvenile (J1) is formed. The J1 molts into J2 stage that hatch from eggs. At the onset of parasitism, the infective J2s penetrate the roots and migrate toward the vascular system of root tissues where they develop permanent feeding sites termed “giant cells.” This feeding site serves as a nutrient source for the growth of J2 and other development stages. The J2 molts to become J3, which develops into J4 stage and then adult females which lay eggs in a gelatinous sac (Caillaud et al., 2008; Jones et al., 2013).

For decades, pre-planting treatment of soil beds with fumigant chemicals has been the primary practice for controlling nematodes in vegetable crops in the southern United States; however, use of fumigants is being restricted because of their broad-spectrum and notoriously toxic effects (Zasada et al., 2010). The phase out of methyl bromide, and high costs and application difficulties associated with other chemical fumigants have led to identification and development of potential alternatives for management of *Meloidogyne* spp. (Hajihassani, Davis, and Timper, 2019; Hajihassani, Rutter, and Luo, 2019).

In recent decades, *Brassica* crops have been recognized for control of root-knot nematodes based on their potential use in biofumigation approaches through producing biocidal compounds such as isothiocyanates, and their ability to inhibit the development and reproduction of nematodes (Johnson et al., 1992; Kirkegaard and Sarwar, 1998; Edwards and Ploeg, 2014). Likewise, cereal cover crops including oats can act as non-hosts or poor hosts to *Meloidogyne* spp., resulting in reduced nematode numbers in soil (Wang et al., 2004), if used in a proper crop sequence program. Oilseed radish (*Raphanus sativus* var. *oleiformis*), white (*Avena staiva*) and black oat (*Avena strigosa*) are cool season annual crops that can be grown in the fall and harvested in the spring in regions with mild winter temperatures. These plant species are considered as potential “trap” or “biofumigant” crops for suppressing nematode populations in soil in an integrated program to lessen the use of fumigant nematicides. However, many of oilseed radish and oat cultivars are susceptible to *Meloidogyne* spp. resulting in an undesired population increase in soil during the growth of the crops. To avoid this, oilseed radish and oats cultivars that are resistant should be identified.

Screening for resistance to *Meloidogyne* spp. in radish (Teklu et al., 2014; O’Neill, 2016; Waisen, 2019) and oat (Tateishi et al., 2011; De Brida et al., 2017; Uesugi et al., 2018) has been investigated and several genotypes (lines) and/or commercial cultivars have been characterized. Research on these crops has led to variable suppression of nematodes, depending upon the plant cultivar and nematode species examined (Steddom et al., 2008; Tateishi et al., 2008; Waisen, 2019). Limited information is available on the response of commercially available oilseed radish and oat cultivars toward major species of *M. javanica*, *M. incognita*, and *M. arenaria*. The purpose of this study was to evaluate the host suitability of oilseed radishes and oats for these nematode species, and assess the extent of variation in nematode penetration and development using known susceptible and resistant cultivars.

## Materials and methods

### Nematode inoculum

Pure cultures of *M. javanica*, *M. incognita* race 3, or *M. arenaria* race 1 were increased on eggplant (cv. Black Beauty) grown in steamed field soil in the greenhouse for two to three months. The nematodes were identified to species using species-specific primers and mitochondrial genes (Marquez et al., 2019). Inoculum for experiments was obtained by recovering second-stage juveniles (J2) of each nematode species from infected roots of eggplant by incubating chopped roots in a misting chamber for five to seven days. The J2 collected from a 38-μm-aperture sieve were stored at 5°C until use. The inoculation densities of J2 for the following experiments were determined from three replicates of 1 ml aliquots of the nematode suspension.

### Plant materials

Nine cultivars of oilseed radish, and one cultivar each of white and black oat were evaluated. Seed of oilseed radish cv. Carwoodi, Cardinal, Final, and Image as well as Tachiibuki oat were obtained from Grassland Oregon, Salem, OR. Also, oilseed radish cv. Concorde, Control, Eco-Till, Karakter and Cannavaro as well as Pratex black oat were obtained from Allied Seed, LLC, Nampa, ID. Tomato cv. Rutgers, an excellent host for all root-knot nematode species, was included in the experiments as a standard host.

### Host suitability assay

This study was conducted from June to October 2018. Seeds of the oilseed radish and oat cultivars were germinated in Miracle-Gro Moisture Control potting mix (The Scotts Miracle-Gro Company, Marysville, OH) in seed trays (Speedling Incorporated, Ruskin, FL). Two-week-old seedlings were transplanted individually into Deepot D40L cells (6.9-cm-diam. × 25.4-cm deep; Stuewe & Sons, Inc., Tangent, Oregon) filled with equal parts (v/v) of steamed field soil and washed sand. After two days, initial inoculum of *M. javanica*, *M. incognita*, and *M. arenaria* were applied to plants by pipetting 1,000 J2 into two depressions made in the soil at the base of the stems. The deepot cells were arranged in a completely randomized design with five replications on support trays (Stuewe & Sons, Inc., Tangent, OR), and plants were maintained for eight week, the time needed for the completion of two generations of *Meloidogyne* spp. Temperature in the greenhouse varied between 25 and 28°C. Plants were watered daily and fertilized once, at two-weeks-after planting with 2-g Osmocote smart-release fertilizer (15-9-12, The Scotts, Marysville, OH). Two separate experiments were conducted for each *Meloidogyne* species.

At the termination of each experiment, shoots of all plants were removed and root systems were washed free of soil. Root systems of each plant were weighed and assessed for severity of galling on a scale 0 to 5 where 0 = no galls; 1 = 1 to 2 galls; 2 = 3 to 10 galls; 3 = 11 to 30 galls; 4 = 31 to 100 galls; and 5 = more than 100 galls (Taylor and Sasser, 1978). Root systems from each plant were cut into 2-cm pieces, and eggs were extracted with a 1.0% NaOCL solution (Hussey and Barker, 1973) followed by the standard centrifugal flotation technique. Eggs were counted using an inverted microscope and total number of eggs per gram of fresh root was determined. The reproductive factor (Rf) of *Meloidogyne* spp. was assessed by dividing the final nematode counts extracted from roots by initial number of J2 inoculated to plants at the beginning of experiment, and used as a measure for the host suitability of oilseed radish and oat cultivars to *Meloidogyne* spp. Host suitability was evaluated as follows: Rf < 0.1, nonhost (highly resistant); 0.1 ≤ Rf < 1, poor host (resistant); 1 ≤ Rf < 2, maintenance host (susceptible); and Rf ≥ 2, good host (highly susceptible).

### Nematode penetration and development assay

This assay was conducted from March to May 2019 using Carwoodi and Eco-Till oilseed radish and Tachiibuki and Pratex oat based on their response to *Meloidogyne* spp. in the host suitability study (Table 1). The seeds were planted in 72-cell plug flats (Johnny’s Selected Seeds, Fairfield, Maine) filled with steamed Miracle-Gro potting mix two to three weeks prior to nematode inoculation. Each two-week-old seedling was inoculated with 200 J2 of *M. incognita* race 3, *M. arenaria* race 1, or *M. javanica* in 1 ml water pipetted into two holes (2-cm deep) made around the stem. The experiment was a completely randomized design with three replicates for each cultivar. It was conducted in a greenhouse at 25±3°C. The plants were watered lightly each day. The infected root systems were collected one, three, and five days after inoculation (DAI) to assess nematode penetration. Three seedlings were randomly selected, washed gently with tap water to remove the soil, soaked in 0.5% NaOCL for 30 sec and then rinse with tap water. The nematode J2 in intact roots were stained by boiling for 30 sec in 30 ml tap water containing 1 ml of 3.5% (wt/vol) acid fuchsin. After staining, the roots were rinsed with tap water and destained by boiling in acidified glycerol for about 1 min. Visualization of stained nematodes was done by placing the root systems in a Petri dish and examination under a stereomicroscope (Stemi 508, ZEISS, Hannover, Germany). The penetration of *Meloidogyne* spp. was examined by counting the total number of J2 stained inside the roots at each sampling day. To examine nematode development and reproduction, three root systems were collected at 28 DAI, stained as specified previously and 20 galls were randomly examined under a stereomicroscope to count females. Swollen females with no egg-mass in the root tissues were considered young females, whereas egg-laying females were considered adult females (Fig. 2). This experiment was conducted twice.

**Table 1.** Reproduction of *Meloidogyne javanica*, *M. incognita* race 3, and *M. arenaria* race 1 on oilseed radish, white (Tachiibuki) and black (Pratex) oats under greenhouse conditions.

	*M. javanica*		*M. incognita* race 3		*M. arenaria* race 1	
Cultivar	Rf^y^	Host status^z^	Rf	Host status	Rf	Host status
Tomato^x^	24.0 a	Good	11.1 a	Good	16.5 a	Good
Cannavaro	22.1 a	Good	2.7 cde	Good	3.1 c	Good
Final	22.0 a	Good	2.5 bc	Good	10.2 b	Good
Control	11.0 b	Good	0.3 e	Poor	1.7 c	Maintenance
Image	8.2 bc	Good	7.7 ab	Good	8.2 b	Good
Eco-Till	7.7 bc	Good	3.5 cde	Good	12.5 ab	Good
Pratex	7.1 bc	Good	3.9 cd	Good	9.7 b	Good
Cardinal	4.4 bc	Good	1.2 de	Good	1.9 c	Maintenance
Tachiibuki	3.6 bc	Good	0.2 e	Poor	0.3 c	Poor
Karakter	1.9 bc	Maintenance	1.1 e	Maintenance	0.9 c*	Poor
Concorde	1.8 bc	Maintenance	1.7 de	Maintenance	2.5 c	Good
Carwoodi	1.0 c*	Poor	0.2 e	Poor	0.2 c	Poor

**Notes:** Data are the mean of two trials with ten replicates. Means followed by the same letter(s) within columns are not significantly different (*P* = 0.05) based on Tukey’s test. The asterisks illustrate the Rf is not different from 1 according to LS-means *t*-tests. ^x^Tomato cv. Rutgers was used a susceptible control to compare nematode reproduction variability on crops; ^y^Rf, reproductive factor = final nematode counts extracted from roots/initial number of nematodes (1,000 J2) inoculated to plants at the beginning of experiment. ^z^Host status was determined as follows; Rf < 0.1, nonhost (highly resistant); 0.1 ≤ Rf < 1, poor host (resistant); 1 ≤ Rf < 2, maintenance host (susceptible); and Rf ≥ 2, good host (Highly susceptible).

A histopatological examination was also conducted to evaluate the response of Carwoodi and Eco-Till radish and Tachiibuki and Pratex oat to nematode infection. Two to three segments 1 to 2 cm long of galled roots from each infected plant were examined at 28 DAI. Root segments, including individual small galls and adjoining portions of non-galled tissue, were fixed in buffered formaldehyde solution for 24 to 48 hr at room temperature. The fixed segments were dehydrated in a graded ethanol series (70 to 100%) using a tissue processor (Excelsior AS, Thermo Scientific, Thermo Shandon Limited, Runcorn Cheshire, UK), and embedded in paraffin. Cross sections 4 to 5 μm thick were prepared using a rotary microtome (RM2125 RTS, Leica Biosystems, Nussloch, Germany), and then mounted on glass slides. The sections were stained with Hematoxylin-Eosin (HE), evaluated microscopically at 40 to 100 × magnifications (Olympus BX43, Olympus, Tokyo, Japan) and images captured using a digital camera (Olympus DP73, Olympus, Tokyo, Japan) (Hajihassani et al., 2020).

### Statistical analysis

Galling severity, egg counts, and Rf from the host suitability test were subjected to one-way ANOVA, while data from the penetration and development assay were analyzed by two-way ANOVA using the PROC mixed models (SAS v. 9.4, SAS Institute, Cary, NC). Data were combined prior to ANOVA because the initial and repeat trials were not significantly different (*P* > 0.1). Plant cultivar and DAI (penetration and development assay) were considered fixed effects, and trial was a random effect. Means were compared using Tukey’s adjustment for multiple comparison test at *P* < 0.05. Least-squares means (LS-means) *t*-tests demonstrating if the mean was significantly different from 0 were used to determine whether the nematode Rf was different from 1 (the base 10 algorithm of 1 is 0). Therefore, single *t*-tests that showed the treatment mean was significantly different from 0 indicated that the Rf deviated significantly from 1 (Hajihassani et al., 2020).

## Results

### Host suitability of oilseed radish and oat for *Meloidogyne* species

**Figure 1:**
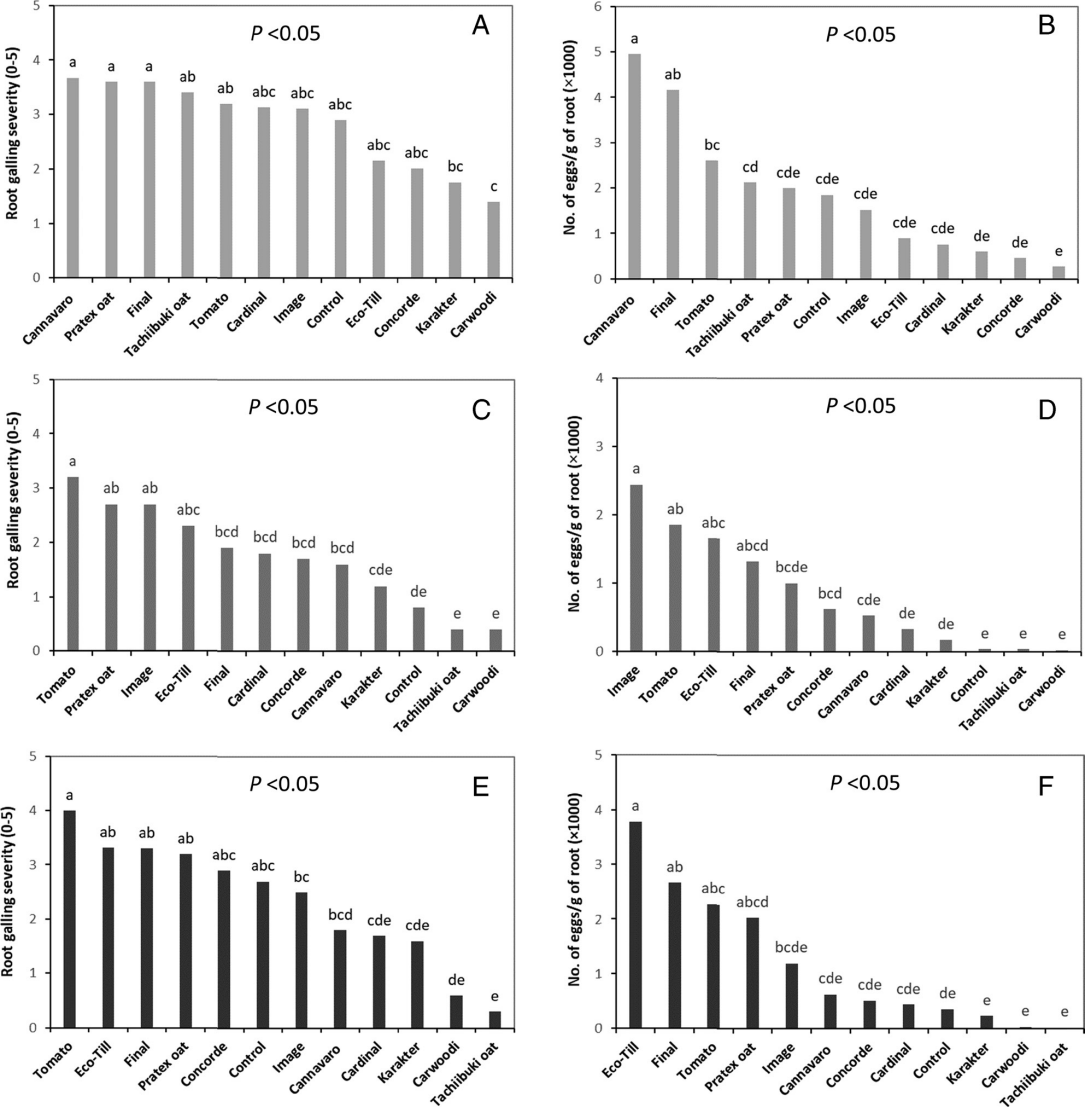
Gall and egg formation of *Meloidogyne javanica* (A and B), *M. incognita* race 3 (C and D), and *M. arenaria* race 1 (E and F) on cultivars of oilseed radish, white (Tachiibuki) and black (Pratex) oats under greenhouse conditions. Tomato cv. Rutgers was used as a susceptible host. Gall severity was assessed based on a scale 0 to 5 where 0 = no galls, and 5 ≥ 100 galls on roots. Data are the mean of two trials with ten replicates. Bars with the same letter(s) are not significantly different based on the Tukey’s test.

Cultivar affected (*P* < 0.0001) all resistance characteristics including gall severity and nematode reproduction. *Meloidogyne javanica*, *M. incognita*, and *M. arenaria* produced varying numbers of galls and eggs in the roots of oilseed radish and oat cultivars (Fig. 1); however, significant differences among the cultivars were infrequent. Root galling severity on the oilseed radish and oat cultivars was in the range of 1.4 (Carwoodi oilseed radish) to 3.7 (Cannavaro radish) for *M. javanica*, 0.4 (Carwoodi) to 2.7 (Image radish and Pratex oat) for *M. incognita*, and 0.3 (Tachiibuki oat) to 3.3 (Eco-Till and Final) for *M. arenaria*. The numbers of *M. javanica* eggs per gram of fresh root ranged from 270 for Carwoodi to 4,946 for Cannavaro. Egg production by *M. incognita* varied from 21 for Carwoodi to 2,436 eggs per gram root for Image. For *M. arenaria*, Tachiibuki (32) and Eco-Till (3,780) had the minimum and maximum number of eggs per gram of root (Fig. 1). All three nematode species reproduced more on tomato than on the oat cultivars and most of the oilseed radish cultivars (Table 1). *Meloidogyne javanica* reproduced on eight cultivars of oilseed radish, and the white and black oats. Although the nematode had an Rf of 1.05 on Carwoodi, the Rf was not different from 1 based on the LS-means *t*-tests indicating that *M. javanica* failed to increase its population on Carwoodi.

The Rf of the three nematode species did not often vary among the cultivars tested. The Rf of *M. incognita* was lower (*P* < 0.05) in Carwoodi and Control oilseed radish and Tachiibuki oat than all other cultivars examined. *Meloidogyne javanica* and *M. incognita* exhibited only slight reproduction on Karakter and Concorde, having Rf’s ranging from 1.0 to 1.9. The Rf of *M. arenaria* was significantly less than 1 on Karakter and Carwoodi oilseed radish and Tachiibuki oat compared to all other cultivars.

### Penetration and development of *Meloidogyne* spp.

**Figure 2:**
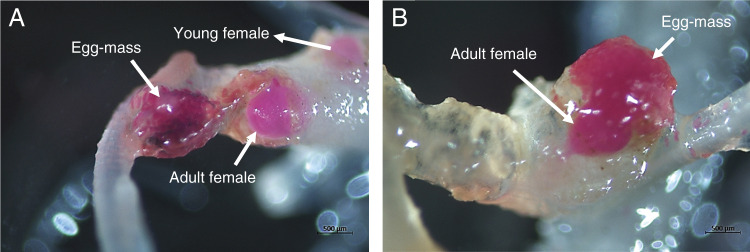
Representative images of roots of oilseed radish cv. Eco-Till (A) and oat cv. Pratex (B) infected with *Meloidogyne arenaria* at 28 days after inoculation.

The penetration of J2 into roots of oilseed radish and oat was only affected (*P* < 0.0001) by DAI, showing that the susceptibility or resistance of the cultivars had no effect on nematode root penetration. At 1 DAI, J2 were observed in the roots of oilseed radish; however, no difference in the numbers of J2 was observed between susceptible Eco-Till and resistant Carwoodi cultivars for *M. javanica*, *M. incognita*, or *M. arenaria* (Table 2). A similar trend in the penetration of the nematodes in roots of susceptible Pratex and resistant Tachiibuki oats was also detected (Table 3). At 3 and 5 DAI, comparable numbers of J2 penetrated the roots of susceptible and resistant cultivars of oilseed radish and oat. Nematode development was studied by counting the numbers of young and adult (egg-laying) females of each nematode species within galled (swelled) roots at 28 DAI. For all three nematode species, greater numbers (*P* < 0.05) of young and adult females were observed in the root tissues of the susceptible cultivar Eco-Till than in resistant Carwoodi (Table 2). Similarly, the total number of young and adult females for *M. incognita* and *M. arenaria* was higher (*P* < 0.05) in the roots of susceptible Pratex (Fig. 2A and B) compared to resistant Tachiibuki oat (Table 3).

**Table 2.** Number of second-stage juveniles (J2), and young and adult (egg-laying) females of *Meloidogyne* spp. in roots of oilseed radish (Eco-Till, susceptible; Carwoodi, resistance) at early-stage of infection (1, 3, and 5 days after inoculation; DAI) and at 28 DAI in the greenhouse.

	J2	J2	J2	Young female	Adult female
Nematode/Cultivar	1 DAI	3 DAI	5 DAI	28 DAI	28 DAI
*M. javanica*					
Eco-Till	8.5 a	19.7 a	30.6 a	20.7 a	17.6 a
Carwoodi	2.7 a	12.3 a	27.0 a	6.6 b	4.2 b
*M. incognita* race 3					
Eco-Till	7.0 a	19.6 a	31.3 a	12.0 a	9.6 a
Carwoodi	3.1 a	12.3 a	27.1 a	3.6 b	1.6 b
*M. arenaria* race 1					
Eco-Till	7.5 a	16.3 a	28.0 a	10.3 a	18.0 a
Carwoodi	5.0 a	21.6 a	23.6 a	3.0 b	1.8 b

**Notes:** Each plant was inoculated with 200 second-stage juveniles of each nematode species. Data are the mean of two trials with six replicates. Means followed by the same letter within columns are not significantly different (*P =* 0.05) based on Tukey’s test.

**Table 3.** Number of second-stage juveniles (J2), and young and adult (egg-laying) females of *Meloidogyne* spp. in roots of oat (Pratex, susceptible; Tachiibuki, resistance) cultivars at early-stage of infection (1, 3, and 5 days after inoculation; DAI) and at 28 DAI in the greenhouse.

	J2	J2	J2	Young female	Adult female
Nematode/Cultivar	1 DAI	3 DAI	5 DAI	28 DAI	28 DAI
*M. javanica*					
Pratex	4.7 a	15.0 a	28.1 a	…^x^	…
Tachiibuki	6.0 a	14.3 a	24.3 a	…	…
*M. incognita* race 3					
Pratex	5.6 a	11.0 a	23.5 a	17.4 a	20.5 a
Tachiibuki	3.6 a	8.3 a	18.0 a	2.3 b	1.6 b
*M. arenaria* race 1					
Pratex	8.0 a	15.3 a	25.3 a	16.0 a	12.3 a
Tachiibuki	6.3 a	14.0 a	29.1 a	3.5 b	2.6 b

**Notes:** Each plant was inoculated with 200 second-stage juveniles of each nematode species. Data are the mean of two trials with six replicates. Means followed by the same letter within columns are not significantly different (*P =* 0.05) based on Tukey’s test. ^x^Pratex and Tachiibuki were susceptible to *M. javanica* and thus not examined at 28 DAI.

**Figure 3:**
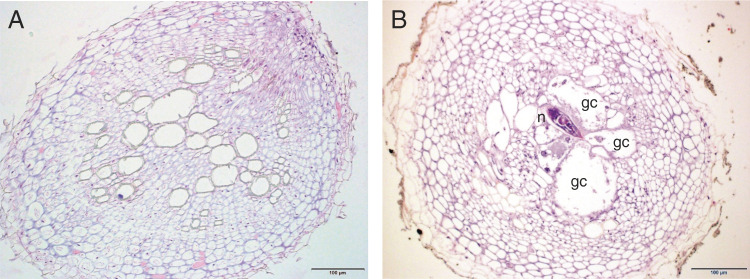
Hematoxylin-Eosin stained cross sections of root tissues of oilseed radish cv. Carwoodi (A) and Eco-Till (B) infected with *Meloidogyne incognita* at 28 days after inoculation. N, female nematode; GC, giant cell.

**Figure 4:**
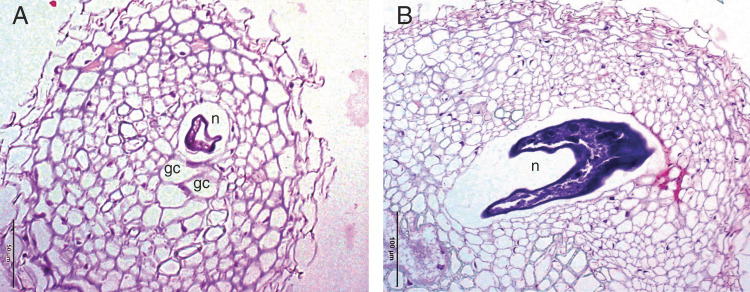
Cross sections of root tissues of oat cv. Pratex (A) and Tachiibuki (B) infected with *Meloidogyne javanica* at 28 days after inoculation. N, female nematode; GC, giant cell.

Cross sections of the root system of oilseed radish and oat infected with all three root-knot nematode species showed morphological variations between susceptible and resistant cultivars. At 28 DAI, more than 96% of the root tissues of resistant Carwoodi (Fig. 3A) and Tachiibuki (not shown) inoculated with *M. incognita* or *M. arenaria* had no sign of feeding site or giant cell formation. In contrast, all the root tissues of susceptible Eco-Till (Fig. 3B) and Pratex (not shown) infected with *M. arenaria* or *M. incognita* had multiple giant cells. We noticed very limited hypertrophy in cortical cells and the vascular cylinder of Carwoodi and Tachiibuki infected with these two nematode species, suggesting that the nematode J2 penetrated the roots but failed to establish giant cells. Multinucleated giant cells were detected in 100% of the root tissues of Eco-Till infected with *M. javanica* (not shown). Cross sections of the root system of *M. javanica*-infected Carwoodi showed degraded giant cells in 5 to 10% of the roots (not shown). Multiple giant cells and severe hypertrophy in the vascular cylinder and cortical cells were detected in the root tissues of Tachiibuki and Pratex oats infected with *M. javanica* (Fig. 4A, B).

## Discussion

Management of plant-parasitic nematodes in vegetable plasticulture or bare-ground systems in the southern US relies heavily on pre-plant fumigation of soil with 1,3-dichloropropene (Telone II), Chloropicrin or metam sodium (Vapam), followed by in-season application of non-fumigant nematicides (Hajihassani, Davis, and Timper, 2019). One of the alternative management methods is the use of nematode-suppressive cover/trap crops to reduce nematode populations in soil. However, the majority of oilseed and oat cultivars have variable levels of susceptibility to root-knot nematodes, and characterization of resistant cultivars could have important implications in root-knot nematode control. Results from this study suggest that differential responses to infection with *M. javanica*, *M. incognita*, and *M. arenaria* exist in oilseed radish and oat cultivars. We determined the susceptibility/resistance of representative oilseed radish and oat cultivars for each nematode species by comparing the nematode Rf. Other resistance characteristics such as root galling severity and low recovery of eggs from root tissues also indicate that only a few cultivars of oilseed radish and oats evaluated exhibited resistance relative to the susceptible tomato.

In this study, *M. javanica* greatly reproduced on most of the oilseed radish and oat cultivars, and only Carwoodi radish was resistant to *M. javanica*. The level of resistance to *M. incognita* varied among oilseed radish and oat cultivars. Control and Carwoodi radish and Tachiibuki oat did not support reproduction and were resistant to *M. incognita*. Results from this study for oilseed radish are consistent with previous studies (Waisen, 2019) reporting several cultivars of oilseed radish are poor hosts to *M. incognita*. We found that the Rf of *M. javanica* and *M. incognita* on Karakter and Concorde radish was slightly greater than 1 and these two cultivars can retain or increase the nematode populations in soil. *M. arenaria* did not infect Karakter and Carwoodi radish and Tachiibuki oat. All other cultivars were susceptible or highly susceptible to the nematode. A greenhouse study in Washington has shown that Carwoodi oilseed radish is resistant to *M. chitwoodi* (O’Neill, 2016). Our results confirmed that this cultivar is also resistant to *M. javanica*, *M. incognita* and *M. arenaria*. Our data on Tachiibuki white oat are consistent with previous research in which few or no eggs of *M. incognita* and *M. arenaria* were detected in the roots of Tachiibuki (Tateishi et al., 2011). Uesugi et al. (2018) reported that a black oat line KH1a significantly reduced the reproduction of different isolates of *M. incognita*, *M. arenaria*, *M. javanica*, and *M. hapla*. In the present study, all three species of *M. incognita*, *M. arenaria*, and *M. javanica* were pathogenic on Pratex black oat.

Resistance to root-knot nematodes can occur prior to infection by reducing nematode invasion and/or penetration into host plants, at post-infection by limiting or containing nematode movement, development, and reproduction in root tissues, and at both the pre- and post-infection stages (Trudgill, 1991; Proite et al., 2008; Hajihassani, Rutter and Luo, 2019). To investigate the initial infection process of three species of root-knot nematodes in oilseed radish and oat, the penetration of J2 into root systems of selected susceptible and resistant cultivars were examined at 1, 3, and 5 DAI. We found no significant differences in the penetration of *M. javanica*, *M. incognita*, and *M. arenaria* between susceptible and resistant cultivars, suggesting that the resistance to these nematodes does not occur at early stages of infection. Tateishi et al. (2011) reported that similar numbers of *M. incognita*, *M. arenaria*, and *M. hapla* penetrated the resistant Tachiibuki oat and a susceptible host. In contrast, greater numbers of young and mature egg-laying females for all nematode species were observed in infected roots of susceptible Eco-Till radish and Pretext oat at 28 DAI, demonstrating that the resistance could act at post-infection stages. In previous research, roots of Tachiibuki oat infected with *M. incognita* or *M. arenaria* had a lower number of mature females compared to a susceptible host at 30 DAI (Tateishi et al., 2011).

The histopathological observations in our study showed that the root structure in the *Meloidogyne*-infected resistant cultivars of oilseed radish and oat were healthy with no or limited signs of feeding site development. The absence of nematodes and giant cells in the resistant cultivars may be attributed to inhibition of juvenile development in the root tissues. In contrast, the root tissues of susceptible oilseed and oat cultivars had several hypertrophied and multinucleated cells due to root-knot nematode development (Cabasan et al., 2014; Hajihassani et al., 2020). Giant cells serve as an important source of nutrients for nematode growth and reproduction. These structures restrict the translocation of water and nutrients from nematode-infected roots to aboveground parts of the plant (Hussey and Williamson, 1997; Caillaud et al., 2008), thereby adversely affecting plant growth.

In summary, the results suggest that development and reproduction of *M. javanica* are reduced on Carwoodi oilseed radish; *M. incognita* on Control and Carwoodi radishes, and Tachiibuki oat; and *M. arenaria* on Carwoodi and Karakter radishes, and Tachiibuki oat. Crop rotations with these resistant cultivars could be beneficial in control of these yield-limiting nematode species. Oilseed radish and oat are not only grown as cash crops but their potential use as suppressive “biofumigant” and/or “trap” crops for nematode management is of great importance. However, special attention to presence of other plant-parasitic nematodes in soil is necessary when cover cropping with oilseed radishes or oats. For example, Grabau et al. (2017) reported that Defender oilseed radish can increase populations of the root-lesion nematode *Pratylenchus penetrans* in soil (Grabau et al., 2017). Pratex oat which was susceptible to all three root-knot nematode species in this study can have positive impact on reducing stubby-root-nematode (*Paratrichodorus* spp.) populations (USDA-NRCS, 2014). The efficacy of these winter-grown cover crops alone or in combination with in-season use of non-fumigant nematicides for the control of plant-parasitic nematodes in vegetable systems are under investigation in field trials in Georgia, USA.
